# High performance workflow implementation for protein surface characterization using grid technology

**DOI:** 10.1186/1471-2105-6-S4-S19

**Published:** 2005-12-01

**Authors:** Ivan Merelli, Giulia Morra, Daniele D'Agostino, Andrea Clematis, Luciano Milanesi

**Affiliations:** 1 Istituto di Tecnologie Biomediche (ITB – CNR), via F.lli Cervi, Segrate (Milano), Italy; 2 Istituto di Matematica Applicata e Tecnologie Informatiche (IMATI – CNR), via de Marini, Genova, Italy

## Abstract

**Background:**

This study concerns the development of a high performance workflow that, using grid technology, correlates different kinds of Bioinformatics data, starting from the base pairs of the nucleotide sequence to the exposed residues of the protein surface. The implementation of this workflow is based on the Italian Grid.it project infrastructure, that is a network of several computational resources and storage facilities distributed at different grid sites.

**Methods:**

Workflows are very common in Bioinformatics because they allow to process large quantities of data by delegating the management of resources to the information streaming. Grid technology optimizes the computational load during the different workflow steps, dividing the more expensive tasks into a set of small jobs.

**Results:**

Grid technology allows efficient database management, a crucial problem for obtaining good results in Bioinformatics applications. The proposed workflow is implemented to integrate huge amounts of data and the results themselves must be stored into a relational database, which results as the added value to the global knowledge.

**Conclusion:**

A web interface has been developed to make this technology accessible to grid users. Once the workflow has started, by means of the simplified interface, it is possible to follow all the different steps throughout the data processing. Eventually, when the workflow has been terminated, the different features of the protein, like the amino acids exposed on the protein surface, can be compared with the data present in the output database.

## Background

Bioinformatics studies complex biological processes *in silico*, both through the analysis of the nucleotide and protein sequences and through the study of the macromolecular structures and interactions. The aim is to elaborate data at different levels of the molecular biology *central dogma*, in order to turn the enormous quantity of information in our possession into real knowledge.

The exponential growth of sequence databases, due to high speed throughput technologies, creates an enormous flow of genomic sequences that must be elaborated. Such sequences have to be carefully investigated to find genes. Once the coding zones have been checked and translated into proteins, these have to be properly analyzed to understand their function [1]. A technology like grid [2], that allows the implementation of high performance calculation, seems to be ideal for integrating huge quantities of heterogeneous data. Using the *Computing Elements *that compose the Italian Grid.it platform, it is possible to distribute the most time-consuming tasks, subdividing the computation in a set of small jobs.

**Figure 1:**
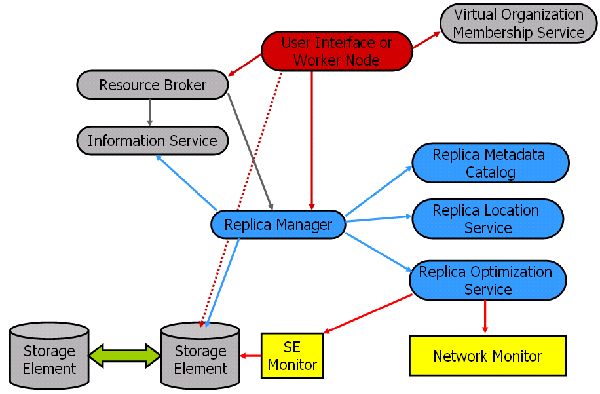
Schema of the Grid.it data management system. Applications access to data through the Replica Manager either directly or through the Resource Broker.

Besides these calculation aspects, Bioinformatics applications need a performing data management system. The Italian Grid.it project offers a set of tools to manage data on a distributed platform [3]. These tools permit the data allocation on different *Storage Elements*, maintaining the coherence between each replica. These databases are efficiently used by the *Resource Brokers*, that coordinate the execution of the different grid jobs in relation to the data location (Fig 1).

## Discussion

The data computation against biological databases [4] is a typical Bioinformatics operation, but the integration of different kinds of analysis is very innovative. The proposed workflow integrates typical applications of the sequence-based Bioinformatics and structural analysis, to give a wide range predictive method for the protein function.

This workflow implements homology comparison of the input proteins against the data present in specific databases, working at different biological levels, starting from the base pairs of the nucleotide sequence to the key residues of the protein surface. In particular, the analysis of the exposed amino acids of the protein surface seems to be very informative about the protein function [5].

### Related Works

Many different web sites offer services of sequence analysis and few web sites nowadays offer services for structural analysis. Important resources are certainly those of the big world Bioinformatics consortia, like the NCBI (*The National Center for Biotechnology Information*), where *BLAST *[6] was developed, and the EBI (*European Bioinformatics Institute*) in which *INTERPRO *[7], a famous set of tools for the protein domains identification, was implemented. From these web sites it is possible to perform calculations on remote clusters and recover the results in short time.

Compared to these services, our workflow has the advantage of integrating data at wide range, analyzing information regarding the protein function starting from the genomic sequence to the exposed residues of the surface, in order to identify few key amino acids. Moreover, the grid technology gives this workflow a notable scalability, that makes it suitable for massive data computation.

From the biological point of view, a number of web sites can be interrogated about the function of specific protein families, but usually it is impossible to compare novel protein sequences to the database. Moreover, it is difficult to correlate information regarding protein families or perform analysis to integrate different kinds of biological data.

For example, the *EF-site *[8] proposes an analysis of some protein families with integrated information about protein surface patterns and amino acids function [9]. However, this approach is not useful for the prediction of protein function, because there is no way to identify structural similarity with the proposed protein families.

The data integration problem has been faced in the *SURFACE *web site [10], that proposes a structural comparison of proteins in terms of amino acids positions [11]. This web site gives an interesting approach to the surface amino acids identification problem, but its prediction possibilities are limited, because users can not submit their own sequences to the analysis pipeline.

The developed workflow, instead, is designed to provide as input a set of sequences and to return data that is immediately integrated into the output database. Users have a prediction method to identify the protein function based on different information, starting from the nucleotide sequence to the similarity of the key amino acids of the protein surface. Moreover, thanks to grid technology, it is possible to coordinate the processing of this information flow by distributing the calculation load in a performing way.

## Implementation

Bioinformatics applications are typically data driven and the workflow technology is useful to coordinate all the computation steps, in particular when it is necessary to integrate different biological aspects. Moreover, Bioinformatics applications usually take a long time to perform: a grid implementation of the related software is a suitable solution to distribute the computation load on a remote platform, while coordinating the whole system from a single web server.

### Workflow description

**Figure 2:**
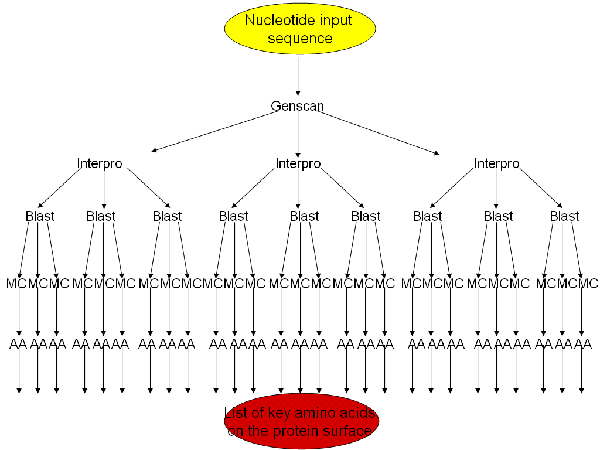
In the first three analysis steps the computation tends to expand, but the information collapse in the definition of few key amino acids.

The first workflow step is the genes identification in the input set of sequences. This task is accomplished using *GENSCAN *[12] that, starting from a set of nucleotide sequences, looks for typical gene patterns, translating the different exons into the corresponding protein sequences. In particular, this tool searches in the nucleotide sequences for the key components of the gene expression like promoters, TATA box sequences and exons patterns. A nucleotide sequence can contain more than one protein sequence: it means that many different jobs are generated from this workflow step (Fig. 2). The homology research proceeds following all the genetic traces present in the input sequences: in this way the range of information is integrated as widely as possible.

In a typical analysis, it is important to identify the protein domains that characterize the macromolecular function, using specific software of domains prediction like *INTERPRO*. This set of tools allows the comparison of different Hidden Markov Models databases with the input sequences to check specific protein domain patterns [13]. Protein domains are very important because they allow the identification of functional sites [14], focusing the analysis on a small dataset of amino acids and neglecting structures that are of minor importance. A protein possesses in general many functional sites that, in the next workflow step, are searched inside a database of protein structures. This passage plays a crucial role, because it expands the homology search to different protein families.

In order to study the three-dimensional structure of a certain domain the workflow performs a *BLAST *against the Protein Data Bank sequence database [15]. The PDB database, hosted by the RCBS (*Research Collaboratory for Structural Bioinformatics*) consortium, contains the atomic coordinates of a large number of protein structures known by crystallography or by nuclear magnetic resonance. Using *BLAST *the workflow identifies a group of structures highly correlated with the input protein domain [16]. The atomic coordinates of proteins, that have strong correlation with the proposed domain, are downloaded from the web site, to create for each domain a set of volumetric models [17]. These models are generated starting from the nuclear coordinates of all protein atoms and consist of a set of three-dimensional matrices that represent different volumetric descriptions of the same protein domain.

**Figure 3:**
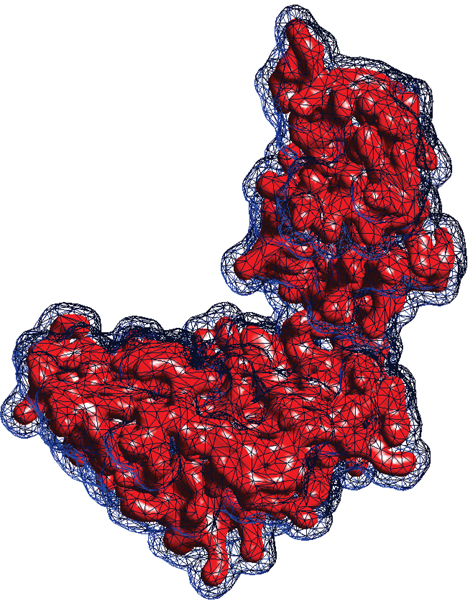
The Lee & Richards surface of the Major Histocompatibility Complex (MHC) macromolecule involved in an interaction with the Human Immunodeficiency Virus (HIV).

The surface is then extracted for each three-dimensional model of the protein domain [18]. This step has fundamental importance to understand which amino acids are effectively exposed to the macromolecular surface and to identify residues that play a key role for the protein function (Fig. 3). The extraction is carried out using the Marching Cubes algorithm [19], that allows to define a triangular mesh for any kind of surface topology starting from its volumetric description.

According to the three-dimensional model, the extracted support mesh represents the so called Lee & Richards protein surface. Through the analysis of each vertex of the protein surface, it is possible to check which amino acids contribute to the external shape of the protein. In this way the information on protein function collapses into the identification of a small dataset of key amino acids.

### Distributed approach

The implementation of this workflow is based on the Italian Grid.it infrastructure. This platform is a network of several *Computing Elements*, that are the gateways for the computer clusters on which jobs are performed, and an equal number of *Storage Elements*, that implement a distributed file system on which databases are stored. The grid is a set of *Resource Brokers*, that are delegated for controlling the execution of the different jobs. To minimize the processing time, jobs are sent to the best calculation site according to the computation load and to the required data transfer. Grid communications are based on a specific *middleware *that relies on the Globus Toolkit [20], a package designed to establish secure connections.

**Figure 4:**
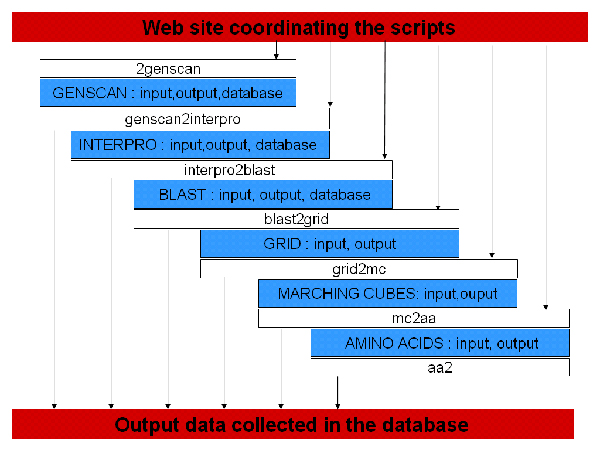
Schema of the script layers: the first layer controls the grid execution of the different analysis steps, the second the management of the I/O.

The workflow management is delegated to a hierarchical structure of scripts that coordinate the execution of the jobs (Fig. 4). At the bottom level there is a set of bash scripts, that are sent to the grid through the *InputSandBox*, a system designed to load files into the *Computing Elements*. These bash scripts set the permissions, retrieve the files containing the databases from the *Storage Elements *and perform the programs according to the environment of the different grid clusters. Each bash script works in close coordination with a JDL script. The latter is written using the Job Description Language and describes the features of the grid job, including the *InputSandBox*, the software requirements and the target databases.

At the middle level a set of perl scripts manage the different steps of the workflow. The first task is to load the input data and divide the computation into a group of small jobs, each one described through a specific JDL script. The jobs are then performed on the grid platform: for each step of the workflow a perl script controls the execution resubmitting the JDL script in case of failure. When a job is successfully completed the perl script takes the output back from the grid, storing the result in a temporary directory. To maintain the output data consistence, each perl script waits until all jobs under its control are correctly finished.

The data flow through the different workflow steps is coordinated by a second layer of perl scripts that parse the output files and redirect the results to the correct directories. This connection layer also deals with the interrogation of the RCBS web site, retrieving the atomic coordinates of the protein from the PDB to perform the structural analysis. Eventually, these scripts are used to store the results in the output database, that consists of a set of tables closely related with the different workflow steps.

## Results

The grid implementation of this system is useful for the distribution of the computational load on a hidden calculation platform. Using this solution, the whole workflow can be coordinated by a single web server, on which the grid *User Interface *software is installed, obtaining a scalable system in relation to the grid performance. The software that gives access to the distributed platform is made up of a set of tools, which ensure secure communications between the grid infrastructure and the web server. Through the grid *User Interface *it is possible to submit jobs, control the workflow state of advancement and retrieve the outputs when the computations have a normal termination or resubmit the jobs again in case of failure.

Due to the use of remote computational resources, the grid communication software must offer an efficient security system. The access to remote clusters is granted by a personal certificate, that accompanies each job to *authenticate *the user. Moreover, users must be *authorized *to job submission by a *Virtual Organization*, a grid community having similar tasks, that grants for them. This procedure is indispensable for maintaining a high security level, but requires time to be accomplished.

**Figure 5:**
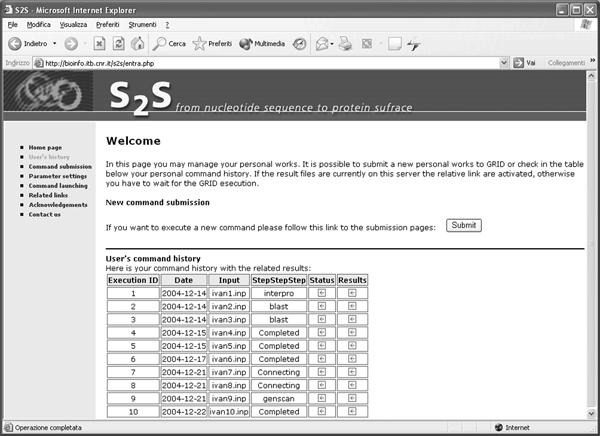
The personal home page of a user after the log in procedure. From this page each user can control the state of advancement of the workflows submitted to the grid platform.

In order to make the workflow user-friendly, a web interface has been developed to manage the whole system, hiding the complexity of the grid infrastructure. This web interface has been developed entirely in PHP and is accessible only through the submission of a password, to maintain a strict control over the grid accesses (Fig. 5). Through this web interface, it is possible to submit a group of nucleotide sequences to the workflow and check the job state of advancement. Eventually, the results can be browsed on the web site, that gives an integrated view of the output data with the information already stored in the database.

The use of the grid infrastructure allows the computation of a huge amount of data, in order to perform genome scale analysis. Several simultaneous users are already supported by the web site, but if requests should increase the web resources shall be improved. Each user can submit sequences in fasta format of several megabytes and the corresponding output ranges from hundreds of megabytes to a few gigabytes of data, that are stored in the output database.

The performance of the workflow is quite difficult to evaluate, because the processing time depends primarily on the total computational load of the grid during the execution of the jobs. According to our tests, if the input dataset is composed of a few megabytes of sequences, the distributed implementation performs as well as a parallel implementation on a dedicated cluster of 8 CPUs with databases shared on a private network. For larger input datasets the distributed implementation is more efficient, because the communication and scheduling procedures become very short in relation to the total computation time. The workflow scalability on the grid infrastructure, in fact, is quite linear, because jobs are not correlated, except in the case of simultaneous accesses to a single replica of a database.

The biological prediction power of the system has to be carefully validated. At present, the possibility to search for homology at different biological levels, starting from the base pairs of the nucleotide sequence to the exposed residues of the protein surface, is unique and very innovative. In particular, the identification of some key amino acids in the macromolecular surface seems very informative on the protein function. Moreover, the workflow output database collects new information for each performed computation, giving a more dynamic predictive sensibility to the system.

## Conclusion

By using the resources of the Italian Grid.it platform, it has been possible to implement a system that integrates numerous Bioinformatics aspects, from the analysis of genetic sequences to the identification of those amino acids that, defining the protein surfaces, are of fundamental importance for the function of the biological macromolecules.

Through different layers of perl scripts it has been possible to coordinate a complex computation system that uses the grid platform for integrating data of different biological databases. On the top of this engine, a web interface was developed for hiding the complexity of the distributed platform. For the success of this project it was necessary to work intensively with databases. The data management in the Italian Grid.it project is founded on a distributed file system, but a software to use DBMS (*Data Base Management System*) over grid is going to be developed. This improvement will certainly make the implementation of Bioinformatics workflows easier.

Grid technology evolution arrives just on time to solve some deeper Bioinformatics problems like the management of huge databases, caused by the exponential growth of biosequences, and the related need for computational resources. Using these high performance platforms, Bioinformatics applications will exceed the present limits, making *in silico *biology capable of solving some of the complex issues of modern life sciences.

## Availability

**Project name: **From Nucleotide Sequence To Protein Surface.

**Project home page: **

**Restrictions: **The access is only for registered Grid.it users having a personal certificate.

## Authors' contributions

IM designed the workflow, ported the system on the grid platform and developed the Web Site. GM was involved in the definition of the protein structural analysis. DD and AC developed the software for protein surface reconstruction. LM contributed to the workflow design and coordinated the project execution. All authors read and approved the final manuscript.
